# Neuropsychological Pattern in a Series of Post-Acute COVID-19 Patients in a Rehabilitation Unit: Retrospective Analysis and Correlation with Functional Outcomes

**DOI:** 10.3390/ijerph18115917

**Published:** 2021-05-31

**Authors:** Davide Antonio Di Pietro, Laura Comini, Lidia Gazzi, Alberto Luisa, Michele Vitacca

**Affiliations:** 1 NeuroRehabilitation of the Institute of Lumezzane, Istituti Clinici Scientifici Maugeri IRCCS, 25065 Brescia, Italy; alberto.luisa@icsmaugeri.it; 2 Scientific Direction of the Institute of Lumezzane, Istituti Clinici Scientifici Maugeri IRCCS, 25065 Brescia, Italy; laura.comini@icsmaugeri.it; 3 Service of Psychology of the Institute of Lumezzane, Istituti Clinici Scientifici Maugeri IRCCS, 25065 Brescia, Italy; lidia.gazzi@icsmaugeri.it; 4 Respiratory Rehabilitation of the Institute of Lumezzane, Istituti Clinici Scientifici Maugeri IRCCS, 25065 Brescia, Italy; michele.vitacca@icsmaugeri.it

**Keywords:** COVID-19, neuropsychological assessment, rehabilitation

## Abstract

Intensive Care Unit delirium, insomnia, anxiety, and frontal/dysexecutive disorders have been described following COVID-19 infection. The aim of this case study was to re-evaluate the neuropsychological pattern in a series of patients with COVID-19 outcomes. We retrospectively evaluated 294 patients admitted to the Istituti Clinici Scientifici Maugeri of Lumezzane (Brescia) (May–September 2020). Neuropsychological assessment was available for 12 patients. We extracted clinical, functional data (FIM and Barthel Index score) and neuropsychological tests (MMSE, Trail making a-b, verbal fluency test, digit span, prose memory test, Frontal Assessment Battery, clock drawing test, Rey–Osterrieth complex figure, Tower of London test). The results were analyzed by Spearman (rho) correlation. Six patients presented dysexecutive alterations even in the presence of normal overall cognitive functioning. Forward digit span score was directly correlated to FIM value at admission (*p* = 0.015) and inversely correlated to delta FIM (*p* = 0.030) and delta Barthel Index (*p* = 0.025). In our experience, subclinical cognitive alterations were present in 4% of patients recovering from COVID-19 pneumonia. The possible correlation between verbal memory and frontal functions, and the degree of functional impairment at admission and its subsequent improvement, underscores the importance of an adequate cognitive evaluation and rehabilitation.

## 1. Introduction

Due to the exponential growth of infected individuals during the global pandemic, the healthcare system transformed our Rehabilitation Institute into a hospital dedicated to post-acute COVID-19 patients. In fact, after severe COVID-19 disease, many patients experience a variety of problems in normal functioning and require intensive rehabilitation to overcome these problems.

Although the clinical presentation is predominantly with respiratory disease, neurological manifestations are increasingly recognized as part of the COVID-19 infection in the context of a poly-systemic syndrome: growing evidence demonstrates the possible involvement of both central and peripheral nervous systems [1]. Even in patients with globally preserved cognitive functions, neuropsychological alterations have been reported, with frequent involvement of attention and frontal functions [2]; deficits of memory, visuospatial and executive functions, with relatively preserved orientation and language, were also described in a series of 13 patients during the post-acute stage of severe COVID-19 [3]. Cognitive malfunctioning, evaluated by the Mini-Mental State Examination (MMSE), appears to be linearly associated with the length of Intensive Care Unit (ICU) stay [4]. In a series of 57 patients, deficits were common in working memory, set-shifting, divided attention and processing speed, without significant association with intubation length [5]. The problem is further complicated by the fact that older patients who experience protracted isolation from their families and their home environment have a further worsening of the cognitive picture. The etiology of these cognitive deficits is currently under study: an association with the severity of the lung affection and potentially restricted cerebral oxygen delivery has been suggested [6].

Physical and rehabilitative medicine and neuropsychology are emerging as major disciplines of post-COVID-19 patient care, with many patients already starting rehabilitation treatment in acute phase and continuing as outpatient after discharge from hospital: indeed, people who have cleared their SARS-CoV-2 infections, but are still not symptom-free (so called “long haulers”), may report continued fatigue, joint and bone pain, palpitations, headaches, dizziness, and insomnia [7].

Although COVID-19 infection poses new challenges and unusual pathological conditions, it is likely that, not unlike other pathological conditions, an early and personalized rehabilitation treatment, conducted both in the early and in the chronic phase, will lead to a greater improvement of the functional outcome parameters [8].

Indeed, across the rehabilitation continuum, neuropsychologists are tasked with determining the best methods of evaluation and treatment based on known cognitive and emotional risks [9]. In acute rehabilitation, and specifically in relation to COVID-19, the task is further complicated and, given the limited understanding of disease-related neurocognitive sequelae and long-term outcomes, a comprehensive understanding of the rehabilitation needs and type of evaluations has not fully emerged [9]. Moreover, it is not clear if neuropsychological alterations are related to long-term functional outcomes in the setting of a Rehabilitation Unit.

The purpose of this case study was to retrospectively evaluate the cognitive pattern of a series of post-acute COVID-19 patients in a NeuroRehabilitation Unit and correlate neuropsychological data with disability and functional recovery scores at admission and discharge.

## 2. Materials and Methods

### 2.1. Patients

We retrospectively evaluated the records of 294 patients undergoing neurological and respiratory rehabilitation for consequences of COVID-19 infection, admitted to the Istituti Clinici Scientifici Maugeri (Rehabilitation Institute of Lumezzane, Brescia, Northern Italy) for recovery in the period May–September 2020.

All patients underwent a rehabilitation program consisting of motor rehabilitation (6 days/week) and, in the last 2 weeks of hospital stay, 150 min/week of occupational therapy (5 days/week). Exercises focused on muscle strengthening (isotonic and isometric exercises) and conditioning, and bed-to-chair mobility, wheelchair skills, pre-gait (sit to stand), bathroom skills, and activities of daily living (ADL) training.

For this study, we considered a series of patients who needed, besides the rehabilitation program, an extensive neuropsychological evaluation during hospitalization. These patients, aged >18 years, had been evaluated in stabilized respiratory condition (PaO_2_/FiO_2_ > 300), with previous diagnosis of COVID-19 infection proven by a positive polymerase chain reaction (PCR) nasopharyngeal swab performed in the acute care hospital. We did not consider for this case study patients with delirium present or antipsychotic therapy in progress.

We obtained informed consent from all patients at admission or from a guardian in the case of severe cognitive dysfunction. The study was approved by the local Ethics Committee (EC 2440, May 2020).

### 2.2. Outcomes

The main outcome was to evaluate if any of the neuropsychological subtests assessing frontal, memory and overall cognitive functions were correlated to the severity of functional impairment at the time of entry and to its expected improvement after the rehabilitation program. The secondary outcome was to describe the type of the neuropsychological alterations found in this series of post-acute COVID-19 patients.

#### 2.2.1. Clinical Data, Disability and Degree of Independence/Need of Assistance in Basic ADLs

We collected the following sociodemographic and clinical data at entry: age, sex, education, and comorbidities by the Cumulative Illness Rating Scale (CIRS—severity index and comorbidity index) [10]. The Barthel Index (BI) assessed patients’ disability at entry and discharge [11]. The total BI score ranges from 0 (maximum level of dependency) to 100 (complete autonomy). A score ≤70 corresponds to severe dependency. The degree of independence and need of assistance in performing basic ADLs was assessed with the Functional Independence Measure (FIM) [12] at entry and discharge: it is an 18-item ordinal scale with 7 levels ranging from 1 (total dependence) to 7 (total independence). The FIM can be subdivided into a 13-item motor subscale (motor-FIM) and a 5-item cognitive subscale (cognitive-FIM). Motor-FIM scores range from 13 to 91 and cognitive-FIM from 5 to 35, with a maximum total score of 126. We also assessed the difference between BI and FIM scores at admission and after the rehabilitation program (delta FIM and delta BI).

#### 2.2.2. Neuropsychological Evaluations

Neuropsychological evaluations were performed by the same skilled neuropsychologist and, at the moment of examination, all patients were negative at SARS-CoV-2 real-time PCR nasopharyngeal swab. Cognitive aspects of the mental function were evaluated by the Mini-Mental State Examination (MMSE) [13]. MMSE scores ≥24 indicate normal cognitive status, lower scores a more or less severe cognitive impairment.

A complete neuropsychological assessment (NA) on cognitive function was also available. In detail, for short-term memory evaluation, we used forward and backward digit span [14]; for long-term verbal episodic memory the story recall test “Anna Pesenti” [15]; to investigate attention Trail Making Test-a and for executive function Trail Making Test-b [16]. To evaluate frontal function, we used the Frontal Assessment Battery (FAB) [17] and phonemic and semantic verbal fluency test [18]; for spatial cognition or non-verbal episodic memory, the Rey–Osterrieth complex figure [19]; to evaluate the visual–spatial factor we used the clock-drawing test [20]; as spatial problem-solving task, we used the Tower of London test [21].

The scores of all neuropsychological tests were adjusted for age and education and were analyzed according to the method of Equivalent Scores (ES), with an Italian calibration when required, as described by Spinnler and Tognoni [22]. The system of ES provides, for each test, a result that ranges from 0 to 4: an ES of 0 constitutes a definitely pathological score and accounts for the lower 5th percentile of a normal distribution; an ES of 1 indicates a border-line score (between the 5th and 20th percentiles); ES of 2, 3 and 4 are considered as fully normal values.

### 2.3. Statistical Analysis

Descriptive data are reported in Table 1 both as mean ± SD and median (25th–75th percentile). For the main outcome, the results of the NA tests were analyzed by Spearman (*rho*) correlation with respect to data at admission and changes in motor function and disability (FIM and BI scales, respectively). Values were considered statistically significant for *p* < 0.05.

## 3. Results

Twelve patients, ten of whom subsequently underwent cognitive rehabilitation, had a neuropsychological assessment after the neurological clinical evaluation: 2 patients because of previous ICU delirium, 2 for worsening of pre-existing cognitive deficits, 3 for attentional deficit or behavioral alterations (disinhibition), and 5 for reported anxiety. As reported by the caregivers, for all patients except the two with pre-existing cognitive deficits, these neurological disorders appeared for the first time in the context of COVID-19 infection. Detailed clinical and neuropsychological data of the selected 12 patients are as follows.

Patient 1 was a 67-year-old male with 13 years of education, suffering from myasthenia gravis with worsening of the motor functions after COVID-19 pneumonia, that required mechanical ventilation and admission to ICU in the acute care hospital. The patient suffered from mild insomnia treated with zolpidem 10 mg in the evening. The neuropsychological evaluation was carried out because of self-reported anxiety at 60 days after the onset of symptoms. The MMSE score (adjusted for age and education) was 26.2/30; the tests of Tower of London, Figure of Rey, and verbal fluency (semantic access) were found to be pathological (equivalent score = 0). The scores obtained on BI and FIM were, respectively, 85 and 102 at admission, and 100 and 124 at discharge, suggesting a preserved functional state.

Patient 2 was a 65-year-old man, with 5 years of education, hospitalized for respiratory failure as a consequence of COVID-19 pneumonia. The patient suffered from ICU delirium and was evaluated 84 days after the onset of symptoms. The MMSE score was 22.9/30, with a final diagnosis of mild cognitive impairment. Neuropsychological assessment showed pathological results (equivalent score = 0) at Figure of Rey, TMT-b and FAB tests. The scores obtained on BI and FIM were, respectively, 20 and 54 at admission, and 80 and 103 at discharge. Despite deficits revealed by the neuropsychological examination, this patient showed moderate disability, but a good degree of independence in motor function.

Patient 3 was a 73-year-old man with 6 years of education, hospitalized for mono-paresis of the lower limb due to hematoma of the psoas muscle, in the course of anticoagulant therapy for pulmonary thromboembolism in the context of the COVID-19 infection. The patient was assessed 60 days after symptoms onset. Premorbid status was described as normal. The MMSE score was 27.3/30; the Figure of Rey test (equivalent score = 0) was found to be pathological. BI and FIM scores at admission were 60 and 99, and at discharge 70 and 104, respectively. These data suggested mild disability and good degree of independence in motor function.

Patient 4 was a 35-year-old man, with 11 years of education, hospitalized for COVID-19 pneumonia outcomes that required mechanical ventilation in ICU. The patient had a history of bipolar disorder, well controlled with lithium therapy, and his cognitive status was described as normal before hospitalization. Neuropsychological tests carried out 45 days after the onset of symptoms showed mild long-term verbal memory deficit (equivalent score = 0); FAB and TMT a, b and b-a scores were at the lower normal limits (equivalent score = 1). The MMSE score was 28/30. The BI and FIM scores at admission were, respectively, 5 (very high disability) and 68, and at discharge 95 and 103.

Patient 5 was a 61-year-old man, with 8 years of education, hospitalized for lower-limb amputation secondary to arterial ischemia of the limb in the course of COVID-19 infection. The patient was mechanically ventilated in ICU. The neuropsychological evaluation was carried out 150 days after the onset of symptoms, and the patient was described as completely self-sufficient before hospitalization. The patient suffered from insomnia treated with lorazepam 1 mg in the evening. Neurological clinical evaluation at admission showed frontal symptoms (disinhibition and deficit of sustained attention). The MMSE score was 24.5/30; neuropsychological assessment demonstrated a deficit of prose retention (“Anna Pesenti” short story). BI and FIM scores were 60 and 89 at admission, and 85 and 112 at discharge, respectively. These data confirmed good ability and degree of independence in motor function.

Patient 6 was a 64-year-old woman, with 5 years of education, hospitalized for critical illness neuropathy after mechanical ventilation in ICU. Neurological and motor status was described as normal before hospitalization. Neuropsychological tests were performed 45 days after the onset of symptoms and demonstrated the presence of frontal behavioral signs (disinhibition, perseveration) with deficits of shifting and attention. The MMSE value was 25.9/30. Alternated phonetic and semantic verbal fluency was pathologically altered (ES = 0). The BI and FIM scores were 0 and 34 at admission, and 79 and 89 at discharge, respectively. This patient had very high disability and impaired motor function at admission, but showed a good recovery after rehabilitation.

Patient 7 was a 69-year-old man, with 5 years of education, hospitalized for severe tetraparesis secondary to critical illness neuropathy, with COVID-19 pneumonia with orotracheal intubation and mechanical ventilation. The patient, evaluated approximately 90 days after symptoms onset, showed a normal premorbid state; during and after ICU stay the patient experienced repeated episodes of excited delirium, treated with low-dose quetiapine (antipsychotic therapy was already discontinued at the time of evaluation). The MMSE score was 24.9/30. The neuropsychological assessment was found to be normal in all subtests. BI score went from 10 (very high disability) at admission to 60 at discharge (severe disability); the FIM score went from 29 (very impaired motor function) at admission to 30 at discharge (persistent high functional limitation).

Patient 8 was a 54-year-old woman, with 12 years of education, hospitalized for gait imbalance after COVID-19 pneumonia which required mechanical ventilation in the ICU. The patient was evaluated 30 days after the onset of symptoms of anxiety with mild depression. The MMSE score was 28/30, and the subtest scores were within the normal limits. The BI score (admission–discharge) went from 60 to 100 and FIM from 72 to 126 (admission-discharge).

Patient 9 was a 52-year-old female, hospitalized for respiratory failure following COVID-19 pneumonia. The patient was evaluated at 150 days after symptoms onset because of self-reported mild cognitive impairment and anxiety. The MMSE value was 25.2/30. The neuropsychological evaluation revealed deficits in long-term verbal memory tests and in the FAB test (equivalent score = 0). BI and FIM scores were unchanged, respectively 85 and 90, both at admission and discharge.

Patient 10 was a 55-year-old woman hospitalized for respiratory failure as a COVID-19 pneumonia outcome, which required mechanical ventilation and tracheostomy. The patient reported attention deficits with moderate anxiety state. Neuropsychological tests were performed 45 days after the onset of symptoms. The cognitive profile was found to be normal overall and all subtests ranged within normal values. The MMSE score was 27/30. BI and FIM values at admission were 85 and 103, and at discharge 100 and 126, respectively. Disability and motor function were already good at entry in this patient.

Patient 11 was an 84-year-old woman, with a previous diagnosis of cognitive impairment, hospitalized for COVID-19 pneumonia and femoral neck fracture. The MMSE was 14.7/30 (indicating severe cognitive impairment). BI and FIM scores at admission were 50 and 72, and at discharge 50 and 73, respectively. This patient was impaired from both a clinical and neuropsychological point of view.

Patient 12 was an 80-year-old man, suffering from dementia, hospitalized for femoral neck fracture and COVID-19 pneumonia outcomes. The score of MMSE was 5.4/30 indicating a very high degree of cognitive impairment; BI was 60 at admission and 70 at discharge; the FIM was 84 at both admission and discharge, suggesting a maintained degree of disability and independence in motor function during the rehabilitative period.

According to MMSE (corrected for age and education), a clinical diagnosis of cognitive impairment prior to COVID-19 infection was confirmed in two cases (patients 11 and 12), who were not further evaluated by complete neuropsychological tests. Clinical and neuropsychological assessments in COVID-19 patients (mean age 64.0 ± 13.7 years, mean education 7.9 ± 3.0 years, 7 males) are summarized in Table 1.

**Figure 1:**
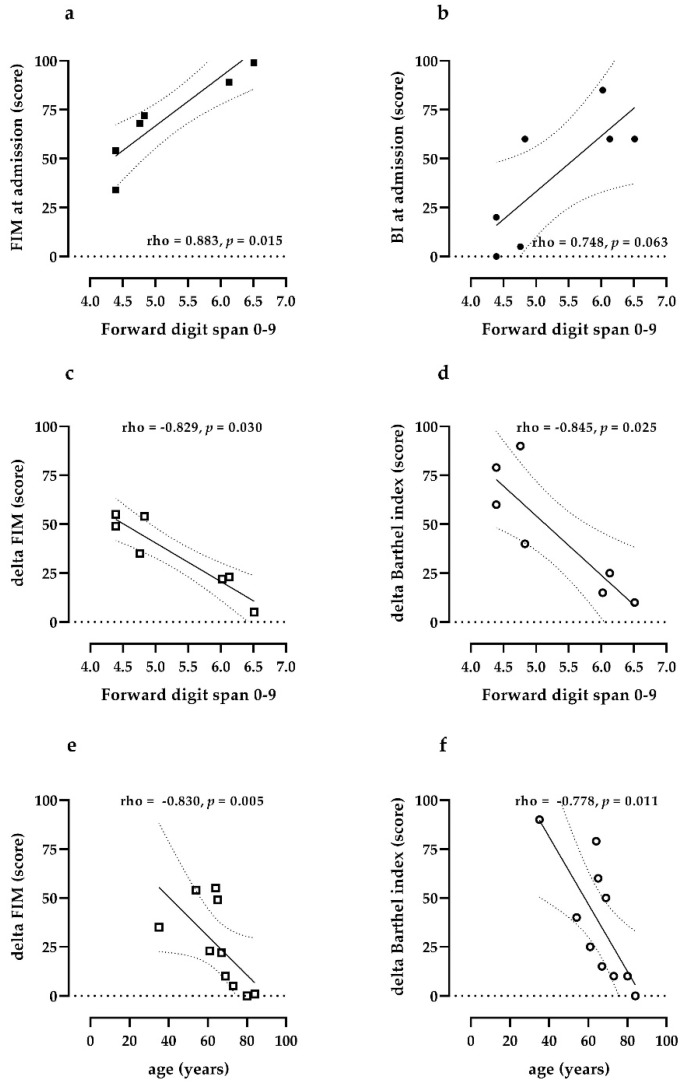
Scatter plot indicating correlation between the forward digit span score 0–9, value of FIM and BI score at admission (panels (**a**,**b**)) and delta FIM and delta BI (panels (**c**,**d**)). Correlations between age and delta FIM (panel (**e**)) and delta BI (panel (**f**)) are also reported.

At admission, this group of patients was characterized by a high grade of disability, as shown by BI (Table 1), and mild motor impairment (FIM scale, Table 1), with mean MMSE within normal limits (27.6 ± 0.6). One patient presented difficulties in accessing the lexicon in a semantic key, 2 patients had visual and spatial disturbances, 4 patients presented deficits in executive functions (reasoning, attention, shifting), 2 patients had difficulty in verbal memory; 2 patients also presented frontal behavioral signs (disinhibition). In one patient (patient 2: male, 65 years), who previously suffered from post ICU delirium, the final diagnosis was of mild cognitive impairment (MMSE = 22.9, Milan Overall Dementia Assessment = 80.7) with deficits in divided attention, abstraction, deductive logical reasoning, and constructive apraxia. We found that the score for the forward digit span test was significantly associated (*p* = 0.015) with FIM score at admission (Figure 1a).

Similarly, bordering on statistical significance, patients with higher BI score at admission tended to reach better scores on the direct digit span (*p* = 0.063, Figure 1b) and FAB tests (*p* = 0.057).

We also observed that delta FIM (*p* = 0.030) and delta BI (*p* = 0.025) were significantly and inversely correlated with the digit span score (Figure 1c,d): in our series, patients with a lower digit span test score, who underwent neuropsychological rehabilitation, had a higher rate of improvement on the functional scale. As expected, both delta FIM (Figure 1e: rho = −0.830 with *p* = 0.005) and delta BI (Figure 1f: rho = −0.778 with *p* = 0.011) were inversely correlated with age.

We did not find a significant association between the absolute score of FIM and BI at discharge and any of the examined neuropsychological tests; there was no significant association between MMSE and CIRS, and the mean MMSE score was similar for patients with and without previous ICU hospitalization.

## 4. Discussion

Our findings suggest that age and alterations in executive functions may be related to the extent of functional damage at admission and to the functional recovery in patients with COVID-19 infection.

Mild-to-moderate cognitive deficits have been described in patients recovering from COVID-19 pneumonia, similarly to those described in patients recovering from acute respiratory distress syndrome (ARDS) due to other pathogens. In a larger series of patients, 70% of ARDS survivors exhibited deficit of attention, speed of reasoning and executive functions; 12% were identified as having post-ICU cognitive impairments by the clinical rehabilitation team, but it was supposed that this might be a significant underestimation, based only on clinical evaluation, in the absence of neuropsychological testing [23].

The evolution of neuropsychological alterations after COVID-19 infection over time is still unclear. The persistence of neuropsychological disorders has been described 3 months after COVID-19 infection: impairment of quality of life, anxiety and depressed mood, sleep disorders, post-traumatic stress disorder, and fatigue were found in up to 23% of patients in a 3 months follow-up of a cohort of patients who recovered from COVID-19 infection [24]. It has been suggested also that these neuropsychological manifestations may cause a substantial socioeconomic burden, as well [9].

The etiology of the cognitive alterations described in COVID-19 patients is also under study. Although previous studies on ARDS from causes other than COVID-19 have not shown any correlation between duration of mechanical ventilation and the degree of neuropsychological impairments [23], other studies have shown, in patients recovering from COVID-19 pneumonia, an inverse correlation between MMSE score and length of ICU stay [4]. This could suggest an added role of the virus as an underlying cause of the cognitive deficits: a direct attack on the CNS due to SARS-CoV-2 has been assumed and is currently being investigated [25,26]. It has also been suggested that underlying long-lasting inflammatory processes may play a role in the cognitive deficits, even in patients who have recovered from COVID-19. The reaction time for the first and second parts of the Continuous Performance Test correlated positively with the C-reactive protein levels in a series of 29 patients, recovered from COVID-19, as confirmed by two consecutive negative nucleic tests [27].

In our experience, 12/294 (4%) patients who recovered from COVID-19 pneumonia needed extensive neuropsychological evaluation based on clinical and neurological evaluation at admission. Excluding two patients with pre-existing severe cognitive impairment, 6 out of 10 patients, whose premorbid cognitive status was normal as reported by relatives, showed signs of frontal or dysexecutive dysfunction, while 2 out of 10 had difficulties in verbal memory. These neuropsychological alterations that we found in post COVID-19 patients are in line with previous reports in the literature. Patients with lower direct digit span and FAB scores tended to have lower functional scores also at admission (FIM and BI); however, these patients seemed to benefit more from rehabilitation, and obtained a greater improvement in the functional parameters examined (FIM and BI). The results of this study provide support for including in the multidisciplinary rehabilitation program an assessment of the patient’s neuropsychological profile besides the motor and cardio-respiratory aspects.

This retrospective study has several limitations, in particular the relatively low number of patients included, probably an indirect result of the limited access to non-essential clinical services (such as the Service of Neuropsychology) during the pandemic, and the lack of a control group of COVID-19 negative patients. Also, we did not have reliable data regarding the premorbid neurological state: we had to rely on the patient’s history and on what was reported by family members. In some cases, we will be able to verify these data as we are planning, on the suggestion of our neuropsychologist, to reassess patients at 8-10 months after discharge.

## 5. Conclusions

In summary, in our series of patients we observed a correlation of verbal and working memory and frontal functions with the degree of functional impairment at admission and its subsequent improvement. Even if, in our series, neuropsychological deficits accounted for a relatively small percentage of post COVID-19 patients, the extent of these deficits is probably under-recognized and patients may benefit from adequate cognitive rehabilitation. In our experience, screening of patients at admission to the Rehabilitation Unit, using simple-to-administer tests assessing frontal and verbal memory functions, could help predict the extent of functional improvement that can be gained with rehabilitation and could help select patients who may benefit from a complete neuropsychological assessment and cognitive rehabilitation. Further studies are necessary to confirm this hypothesis.

## Figures and Tables

**Table 1:** Clinical characteristics (*n* = 10) and NA complete assessments (*n* = 7). Scores of neuropsychological tests were adjusted for age and education.

**Clinical characteristics (*n* = 10)**			
heading	Mean ± sd	Median (25th–75th percentile)	Normal value
Age, years	64.0 ± 13.7	65 (54–73)	
CIRS 1, severity index	2.2 ± 0.5	2.0 (1.8–2.9)	Lower scores (best)
CIRS 2, comorbidity index	5.6 ± 2.5	5.0 (5.0–8.0)	Lower scores (best)
Barthel Index at admission, score	41.0 ± 29.5	55 (9–60)	0 (worse)–100 (best)
Barthel Index at discharge, score	78.9 ± 16.8	80 (68–96)	0 (worse)–100 (best)
Delta Barthel Index, score	37.9 ± 31.1	33 (10–65)	
FIM scale at admission, score	70.3 ± 25.1	72 (49–92)	0 (worse)–126 (best)
FIM scale at discharge, score	95.7 ± 26.0	103 (81–115)	0 (worse)–126 (best)
Delta FIM, score	25.4 ± 21.7	23 (4–50)	
Previous ICU, *n* (%)	6 (50)	--	
Mean ICU stay, days (6 patients)	26.1 ± 10.2	12 (9–51)	
Normal premorbid state, *n* (%)	10 (83)	--	
Symptoms duration, days	75.0 ± 42.4	60 (45–105)	
**NA complete assessments (*n* = 7)**			
Forward Digit span 0–9	5.13 ± 0.95	4.8 (4.4–6.1)	≥4.26
Backward Digit span 0–9	3.94 ± 0.57	4.0 (3.6–4.4)	≥2.65
Story test (early recall), *z*	−0.58 ± 1.11	−1.2 (−1.4–0.3)	<−2
Story test (late recall), *z*	0.24 ± 1.60	−0.7 (−1.5–0.9)	<−2
TMT-a, score	32.63 ± 22.41	30 (18–54)	≤128
TMT-b, score	96.17 ± 55.22	81 (56–141)	≤295
FAB, score	14.58 ± 2.22	15 (12–16)	≥13.5
Phonemic verbal fluency test	24.10 ± 6.60	23 (20–30)	≥17.77
Semantic verbal fluency test	38.11 ± 6.97	38 (33–44)	≥28.34
Rey–Osterrieth complex figure test	30.14 ± 7.81	34 (20–36)	≥28.87
Clock drawing test *	12.40 ± 2.70	14 (10–15)	≥7.57

Legend: Cumulative Illness Rating Scale (CIRS), Motor Functional independence (FIM), Trail Making Test (TMT), Frontal Assessment Battery (FAB). * available in 5 patients.

## Data Availability

The data presented in this study are available on request from the corresponding author.
